# Incidental *FOXL2* mutated adult granulosa cell tumour of the ovary with thecoma-like foci

**DOI:** 10.1007/s00428-022-03452-y

**Published:** 2022-11-18

**Authors:** Anne Kristin Fischer, Birgid Schömig-Markiefka, Carina Heydt, Dominik Ratiu, Peter Mallmann, Jörn Meinel, Reinhard Büttner, Dietmar Schmidt, Alexander Quaas

**Affiliations:** 1 Institute of Pathology, University of Cologne, Kerpener Str. 62, 50937 Cologne, Germany; 2 Department Od Gynaecology, University of Cologne, Kerpener Str. 62, 50937 Cologne, Germany; 3 Medical Care Center for Histology, Cytology and Molecular Diagnostics Trier, Max-Planck-Straße 5, 54296 Trier, Germany

**Keywords:** Adult granulosa cell tumour, Thecoma-like foci, *FOXL2* mutation, Functioning ovarian neoplasms, Reticulin staining

## Abstract

We report on the incidental finding of a *FOXL2* mutated adult granulosa cell tumour of the ovary with thecoma-like foci, a rare entity recently described by Jennifer N. Stall and Robert H. Young in a series of sixteen cases in 2019, displaying features differing from conventional adult granulosa cell tumour. Our aim is to specify the morphologic and molecular particularities of this presumably underrecognized finding, with a short presentation of the typical clinical context. Awareness of this rare and challenging neoplasm with indeterminate clinical course is crucial in routine diagnostics.

## Introduction

Granulosa cell tumour of the ovary was first described by Rokitansky in 1855 [1]. A further histopathological discrimination and subcategorization of different, occasionally mixed forms of sex cord-stromal tumours followed in the subsequent decades. Adult granulosa cell tumour of the ovary with thecoma-like cells is a very rare, presumably underrecognized finding. It is currently not listed as a separate entity in the WHO classification of 2014. Considering the 2019 published series of sixteen cases by Jennifer N. Stall and Robert H. Young [2], this entity appears to be distinguished by particular clinical and morphologic features.

## Case report

A 67-year-old obese woman (BMI 29.4) presented with postmenopausal uterine bleeding in December 2021. Clinical examination showed a high endometrium (3 mm). Adnexa were not assessable via ultrasonography because of intestinal overlay and obesity. Consecutive endometrial biopsy confirmed atypical endometrial hyperplasia. Clinical anamnesis revealed recent spontaneous four-level-deep vein thrombosis, nicotine abuse, arterial hypertonia, and hypercholesterinaemia. Reportedly, the father suffered from thrombophilia with deep vein thrombosis and pulmonary emboli. The last gynaecological examination had been executed 15 years ago. To treat coagulopathy with risk of thrombosis and pulmonary emboli, the patient received antithrombotic and antihypertensive medication (rivaroxaban, candesartan, and aspirin). She was treated with gestagens for 1 year, then hysterectomy with bilateral adnexectomy was performed.

Macroscopy showed an enlarged right ovary measuring 4.0 × 2.5 × 1.3 cm, and a normal-sized left ovary of 2.0 × 1.8 × 1.3 cm.

**Fig. 1:**
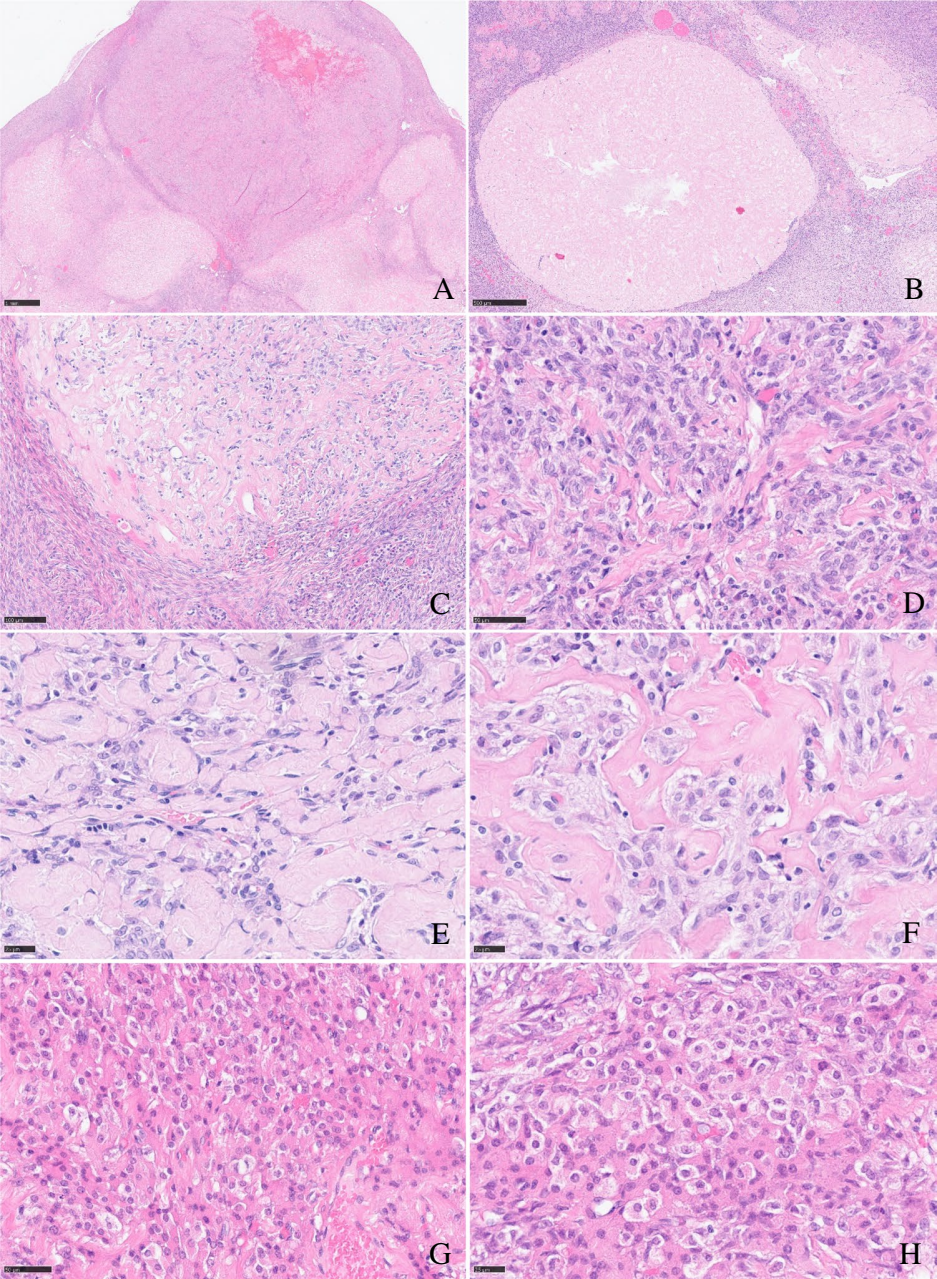
**a**, **b** Tumour overview. Typical multinodular growth pattern of granulosa cell tumours with thecoma-like foci: One big tumour nodule bulges the ovarian surface (**a**), smaller interspersed sclerosing tumour nodules, surrounded by ovarian stroma (**b**). **c** Tumour heterogeneity: Alternating spindle cell areas with typical granulosa cell morphology and sclerosing tumour areas of low cellularity. **d** Storiform growth pattern with typical granulosa cell morphology with irregular or angulated nuclei with occasional grooves. **e**, **f** Sclerosing tumour areas with broad hyalinized collagen bundles. **g**, **h** Thecoma-like foci. Nesting epithelioid, luteinized tumour cells with ample eosinophilic cytoplasms and central round, monomorphic nuclei

**Fig. 2:**
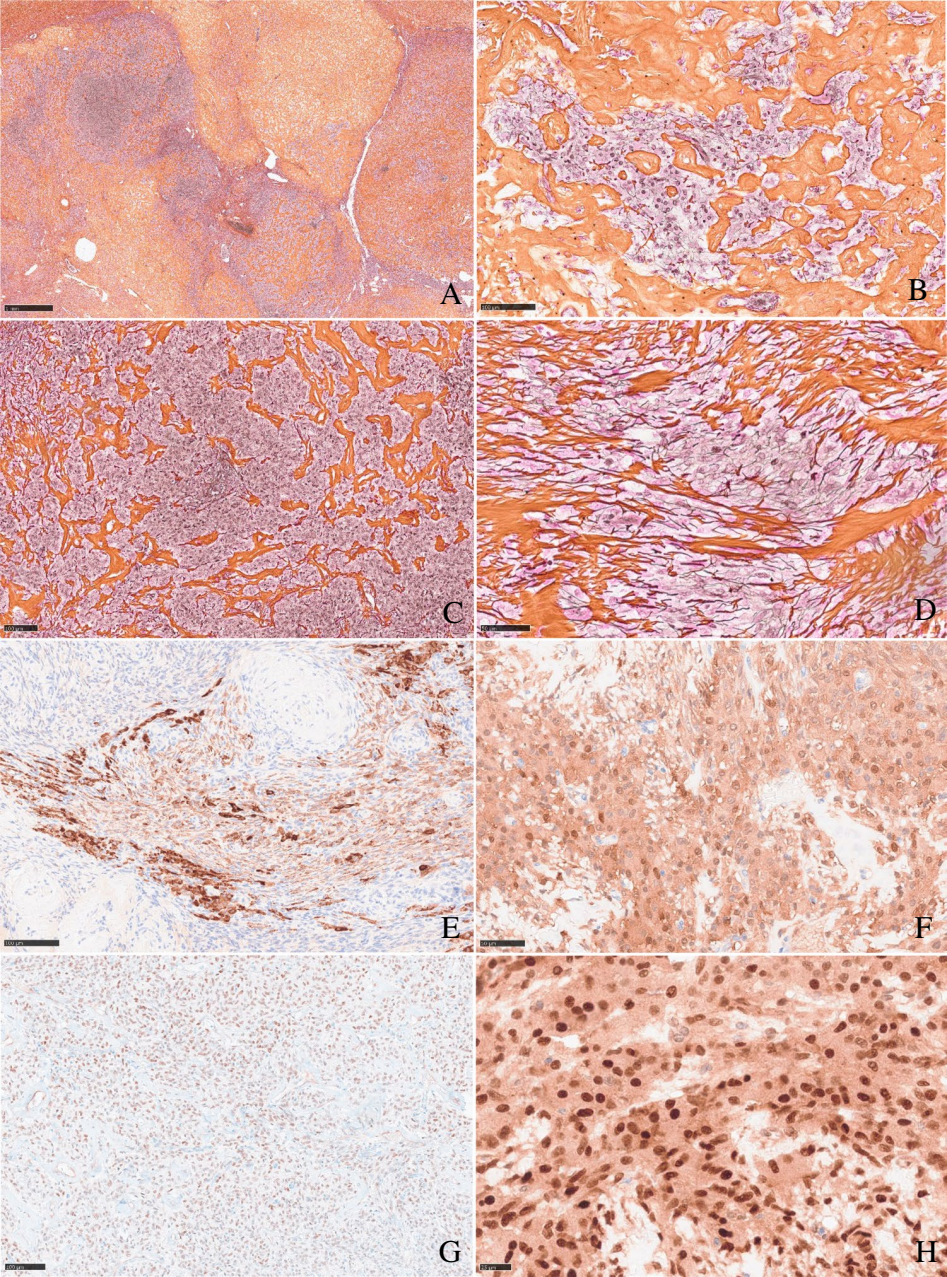
**a** Tumour overview: Multinodular architecture, highlighted by reticulin fibre staining. **b**, **d** Thecoma-like granulosa cell component, reduced reticulin staining. **c** Granulosa cell tumour component, reduced reticulin staining. **e** Patchy inhibin staining; interspersed tumour cells show a strong cytoplasmatic reactivity. **f** Calretinin positivity in thecoma-like granulosa cell component, tumour cells with epithelioid, luteinized morphology. **g** Weak nuclear progesterone staining. **h** Strong nuclear SF1 expression in thecoma-like areas, luteinized epithelioid granulosa cell tumour areas

Histology showed endometrial mucosa with typical atrophic, polypoid, and oedematous changes consistent with longstanding gestagen therapy. Only one small focus of sparse atypical endometrial glands had remained. H&E routine examination of the right ovary revealed conspicuous multinodular ovarian stroma with partial excessive sclerosis, and one embedded nodule of enlarged epithelioid cells resembling luteinized theca cells. Reticulin fibre component was strongly reduced in this cell nodule, lacking the typical single cell ensheathment around true theca cells. Complete material workup of both ovaries showed a left ovary without any suspect features. Low-resolution examination of the right ovary revealed one bigger stroma nodule measuring about 1 cm, bulging the ovarian cortex (Fig. 1a, b). High-resolution examination displayed a large sclerosing stroma with a storiform or irregular pattern of broad hyalinized collagen fibres and interspersed granulosa cells with inconspicuous angulated nuclei, occasionally with nuclear grooves (Fig. 1d), and more cell-rich, spindle cell-like areas remnant of smooth muscle cells in leiomyomata (Fig. 1c). Small nests of reticulin-poor interspersed thecoma-like cells were depicted, displaying ample eosinophilic or clear cytoplasms and centrally located, round nuclei with fine granular chromatin without nucleoli or mitotic figures (Fig. 1g, h). Immunohistochemistry showed a marker profile of sex cord stroma tumour, equally expressed in both the (sclerosing) granulosa cell and the thecoma-like tumour cell component. Immunoreactivity for steroid hormones (steroidogenic factor 1 (SF1), oestrogen, and progesterone receptor (only very weak)) was seen. Also, WT1, calretinin, and inhibin (Fig. 2a–d) were positive. A component of desmin-positive, presumably myofibroblastic, spindle cells surrounded the bulging tumour nodules, but the tumour cells themselves did not show desmin expression.

At first it was unclear if the above-mentioned haematoxylin and eosin (H&E) morphological features had to be contextualised as regressive changes of ovarian parenchyma, considering patient age and previous antihormonal therapy. However, the bulging nodular aspect and especially the conspicuously reduced reticulin staining in both granulosa and theca cell-like areas evoked the idea of an unusual ovarian neoplasm, lacking typical features of a clearly defined group member of sex cord-stromal tumours. Complete workup of both ovaries, and additional reticulin and immunohistochemical staining finally confirmed a steroid-producing tumour of the sex cord-stromal tumour family.

We primarily discussed adult granulosa cell tumour with unusual features, particularly considering interspersed thecoma-like areas and excessive sclerosis. Differential diagnoses were (A) granulosa theca cell tumour, (B) sclerosing sex cord-stromal tumour, and (C) fibrothecoma with granulosa cell aspects. Neither the clinical context, especially the advanced patient age, nor the distinct morphology lacking large epithelioid tumour areas matched with sclerosing sex cord-stromal tumour. The dearth of reticulin fibres in the circumscribed thecoma-like areas virtually excluded thecoma with granulosa tumour cell elements or granulosa theca cell tumour. Referring to a series of adult granulosa cell tumours of the ovary with thecoma-like foci published in 2019 by Jennifer N. Stall and Robert H. Young [1], we thought that our finding was mostly consistent with this diagnosis. Reference pathology finally confirmed the diagnosis of an incidental adult granulosa cell tumour of the ovary with thecoma-like foci (Table 1).

**Table 1:**
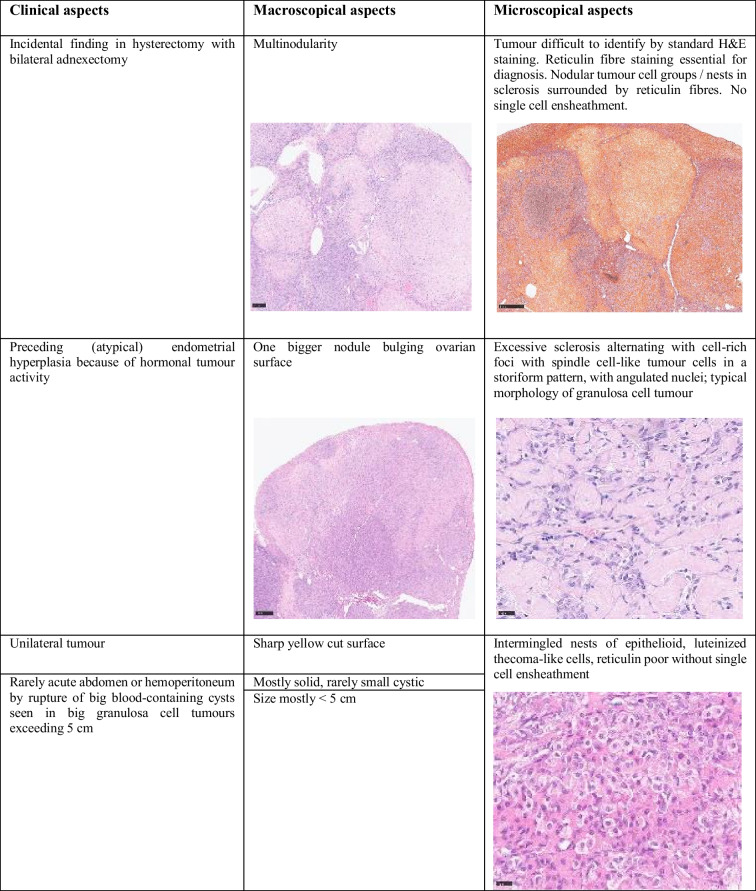
Typical clinical, macroscopic, and microscopic aspects of incidental adult granulosa cell tumour with thecoma-like cells.

## Diagnosis

Incidental adult granulosa cell tumour of the ovary with thecoma like-features (FIGO stage IA).

## Molecular examinations

We performed broad additional molecular analysis on molecular variants, fusions, splice variants, microsatellite status (MSI), and tumour mutation load via next generation sequencing (NGS) using TruSight Oncology 500 (TSO500) assay. Parallel sequencing libraries were established through enrichment of RNA and DNA achieved by hybrid capture procedure. Sequencing was performed via sequencing platform NextSeq (Illumina). Bioinformatic analysis was executed via TSO500 local app (Illumina). Data evaluation was based on the following thresholds: allele frequency (AF) 5%, sequencing depth/coverage 200 × after duplication, tumour cell content 95%. We found a highly specific activating *FOXL2* c*.*402C $\to$ G (p.C134W) point mutation, encoding Forkhead box L2 gene transcription factor that is involved in ovarian development and function. It is a potential tumour suppressor, regulating apoptosis, cell cycle progression, and cell adhesion. Regulation of gene expression by FOXL2 involves interactions with other transcription factors such as nuclear receptors and the SMAD family of transcription factors. *FOXL2 (C134W)* mutation is highly frequent in adult granulosa cell tumours (95–97%), increasing the induction of aromatase, a known target of FOXL2. This mutation also confirms the diagnosis on the molecular level. To our knowledge, it is the first time that *FOXL2 (C134W)* mutation was proved in incidental adult granulosa cell tumour of the ovary with thecoma-like features.

## Discussion

Granulosa cell tumour of the ovary is often described as an incidental finding in only one ovary after hysterectomy with bilateral adnexectomy in the setting of atypical endometrial hyperplasia with bleeding, rarely associated with endometrial carcinoma [2]. Morphologically, the tumour is characterized by (a) a *multinodular growth pattern*, often with (b) *one bigger bulging nodule*, and (c) a *sharp yellow cut surface*. Microscopical characteristics comprise (d) *areas of extensive hyalinized sclerosis* with (e) *compressed strands of inconspicuous small spindle cells* with angulated nuclei, and (f) *intermingled nests of epithelioid thecoma-like cells* with pale eosinophilic cytoplasms and small, round, or angulated nuclei with nuclear grooves [2] (Fig. 1, Table 1). Immunohistochemistry corresponds to typical sex cord-stromal tumour profile (positivity for SF1, calretinin, inhibin, vimentin, often FOXL2; often oestrogen and progesterone receptor expression; and negativity or weak patchy staining for keratin, negativity for EMA, PAX8, and PAX2) (Fig. 2). Most important and diagnostically relevant is the reticulin staining [2]. Significant reticulin loss highlights small nests of thecoma-like cells embedded in extensive sclerosis. In contrast to true thecoma cells, these thecoma-like nests do not present the typical argyrophilic single cell ensheathment classically observed in thecoma. A prominent fibre component in sclerotic areas may be seen, but reticulin staining is strongly reduced or lost in both in the granulosa cell and the thecoma-like foci, demonstrating the characteristic nodular tumour architecture. The reticulin staining is a simple, helpful, and a quick method to foreground features of a tumour in suspect-appearing sclerotic ovaries.

Adult granulosa cell tumour (AGCT) belongs to the group of pure sex cord-tumours (deriving from granulosa or Sertoli cells). Juvenile granulosa cell tumour (JGCT), arising in children and young adults, represents a clinico-pathologically distinctive entity that differs from AGCT in many aspects. In opposition, the current WHO classification lists the group of pure stromal tumours, originating from stromal cells (e.g. fibroma, thecoma), and the group of mixed sex cord-stromal tumours with aspects of both (e.g. Sertoli-Leydig cell tumour). However, as broadly described in the literature since the 1930s by Traut et al. [3], mixed forms between AGCT and fibrothecoma arise, with varying compounds of both entities in one neoplasm. The final tumour classification as ACGT, fibrothecoma or a mixed form, depends on the respective degree of different tumour cells. In 1968 and 1977, Norris et al. [4] and Stage et al. [5] described tumours with a percentage of granulosa cells between 10 and 50% as granulosa cell tumours or mixed granulosa theca cell tumours. Young et al. considered fibroma/thecoma with a granulosa cell component less than 10% as benign “fibrothecomas with minor sex cord elements” [6]. Tumours with more than 50% of granulosa cells were supposed to correspond to AGCT. In a more recent review (2014), Oliva and Young classified tumours with more than 10% granulosa cells on a fibrothecomatous stroma as granulosa cell tumours [7].

Generally, ACGT occurs predominantly in postmenopausal women (peak incidence 50–55 years) [8–10]. As the most common functionally active, oestrogenic ovarian tumour, it often presents with uterine bleeding caused by tumour-induced (atypical) endometrial hyperplasia, endometrial polyps [11–13], and rarely by endometrium carcinoma [1, 6, 14]. Only occasionally an androgenic or progestogenic effect is observed. There are only a few reported cases in the literature describing AGCT arising from extraovarian tissue, mainly from the broad ligament [15]. AGCT is a low malignant tumour with potential of (often late) recurrence and metastasis in 20–30% even in early stages [16, 17], sometimes decades after the initial diagnosis [11, 14, 18]. Documented 10-year overall survival varies. Older studies report survival rates from less than 60% to 90%. However, a recent study from 2016 by McConechy et al. found that 10-year overall survival was identical to the general population, with a median time of recurrence of 7.2 years [19]. A study from 2015 by Wilson et al. reported a relapse of one-third of stage I tumours with a medium relapse time of 12 years [20]. Several long-term studies from the late 1970s and early 1980s also demonstrated late progression [9, 10, 21, 22], with the latest documented relapse after 37 years [19]. It is presumed that lower survival rates might be due to the inclusion of misdiagnosis (in one study, in more than 50% of the cases [12]). Histopathologic errors include metastatic lobular breast carcinoma, malignant melanoma, epithelioid mesothelioma, as well as small cell carcinoma of the hypercalcaemic type (OSCCHT) and clear cell carcinoma [7, 13], which are considered as the most important, albeit rare, differential diagnosis of AGCT [11]. Juvenile granulosa cell tumour must be mentioned as differential diagnosis with a more favourable curse. Luteinized subtype of AGCT can be mistaken as a steroid cell tumour. Again, reticulin staining is helpful, highlighting single cell ensheathment of the latter [13].

In 2008, Shah et al. demonstrated *FOXL2* c*.*402C $\to$ G (p.C134W) mutations in 95–97% of pure adult granulosa cell tumours [23], considered as a good molecular discriminator in diagnostically uncertain cases [24]. *FOXL2* encodes Forkhead Box L2 (FOXL2), suggested to act as a tumour suppressor in granulosa cells mediating apoptosis. Pathogenic *FOXL2* mutation is discussed to cause imbalances in the transforming growth factor β (TGFβ)-signal pathway via impaired interaction with SMAD transcription factors [25, 26]. However, especially in mixed forms with aspects of fibrothecoma, *FOXL2* mutation can be negative [2]. Nolan et al. found *FOXL2* mutations in six of twelve mixed granulosa theca cell tumours with a granulosa tumour cell component greater than 30% [27]. In five cases, *FOXL2* mutations were detected via microdissection in both the granulosa cell and the thecoma cell component. McClugagge et al. reported *FOXL2* mutations in so called “cellular mitotically active fibromas with epithelioid nodules” but not in cellular fibromas [24], and Shah et al. described a *FOXL2* mutation in a “thecoma with minor granulosa cell component” [23]. These findings evoke the question if these cases, in fact, corresponded to mixed forms of granulosa cell tumour with a significant and predominant fibrothecomatous component, further supporting the consideration that on a molecular basis, mixed tumours with a minor percentage of granulosa cells might be more consistent with granulosa cell tumour.

Prognostic factors are difficult to determine. To date, only the International Federation of Gynaecology and Obstetrics (FIGO) stage appears to be the single reliable and reproducible factor to estimate risk of recurrence or metastatic dissemination [18, 28–30]. Ninety percent of AGCT present at FIGO stage I with good prognosis (90% 5-year overall survival); stage IV is a rarity [12, 31]. Some studies discuss tumour size as a possible risk factor [14, 17]. Tumours up to 5 cm in diameter were shown to have far better overall survival than bigger tumours from 6 to 15 cm, 10 to 15 cm [14], or 13.5 cm [17]. Bigger tumours tended to be less solid but more cystic and haemorrhagic, with an elevated risk of rupture and haematoperitoneum, hence an increased danger of dissemination and recurrence. In contrast to that, other studies did not find a correlation between tumour size and risk of recurrence, but between tumour stage and elevated mitotic rate [12, 31]. On a molecular basis, one extended Finnish study demonstrated a correlation between long-term, disease-free survival and high-level, zinc-finger transcription factor GATA4 and human epidermal growth receptor HER2 expression [32], and FOXL2 [33], as well as nuclear atypia [34]. This was already described before in 2005 by Anttonen et al. [35, 36] and by Leibl et al. [37]. GATA4 interacts with SMAD3, a member of TGFβ signal cascade. The impact of “nuclear atypia” appears questionable, since other studies describe AGCT with bizarre, “symplastic” nuclei as a degenerative phenomenon without impact on prognosis [6, 13].

Low tumour stage IA requires mere surgical resection with hysterectomy and bilateral salpingo-oophorectomy. If young patients wish to have children, unilateral salpingo-oophorectomy is sometimes possible. A standard therapy for higher tumour stages (IC–IV) or intraoperative rupture does not exist. Defined risk factors for disease relapse are unknown, apart from tumour size and spontaneous or intraoperative rupture. Age at diagnosis does not appear to play a role. Pelvic and retroperitoneal lymphonodectomy and omentectomy may appear obligative in certain cases, adapted to treatment of epithelial ovarian cancer. However, lymph node metastasis and haematogenous spreading is a very rare finding; AGCT rather seeds via peritoneal dissemination [37]. A recent study from 2019 did not find a benefit in lymphonodectomy on overall survival, but a negative effect in surgical outcome, independent from tumour stage [31]. Most tumours recur in the pelvic or abdominal region, seldomly in the liver and bone [20]. Chemotherapy for recurrent and disseminating tumours [38] is platinum or taxane based, possible combined regiments comprise bleomycin, vinblastine or alternatively etoposide, and cisplatin (BVP/BEP) [20, 39]. Furthermore, antihormonal therapies with luteinizing hormone-releasing hormone (LHRH)-antagonists, tamoxifen, aromatase inhibitors, gonadotropin-releasing hormone (GnRH)-analogist leuprorelin, or the progesterone derivate megestrol show good responses in recurring oestrogen secreting tumours [20, 40]. Other modern targeted therapies are considered, especially trials with bevacizumab as an inhibitor of vascular endothelial growth factor [41–44], and the tyrosine kinase inhibitor, imatinib [45]. EGFR targeting may be considered in a subgroup of EGFR positive tumours [37, 46]. Radiation therapy can be an option if surgery is not possible.

## Conclusion

The newly described *adult granulosa cell tumour with thecoma-like foci* is a rare, not easily detectable ovarian neoplasm of low malignant potential. Confronted with endometrial hyperplasia or carcinoma in pre- and postmenopausal women, both clinicians and pathologists should be aware of this unusual cause of pathophysiological changes by unclear hormonal activity. Smaller nodules in the ovaries should undergo attentive observation during radiological and macroscopic examination of the inner genital organs, even if the clinical concern is fixed on the endometrium. Considering the capacity of recurrence and metastasis of adult granulosa cell tumours in up to one-third of cases, it is crucial not to overlook this finding because of a macroscopic sample error or a benign misdiagnosis in the broad spectrum of sex cord-stromal tumours.

## Abbreviations

*AF*: Allele frequency
*AGCT*: Adult granulosa cell tumour
*BEP*: Bleomycin, etoposide, cisplatin
*BVP*: Bleomycin, vinblastine, cisplatin
*EGFR*: Epidermal growth factor receptor
*FIGO*: International Federation of Gynecology and Obstetrics
*FOXL2*: Forkhead Box L2
*HER2*: Human epidermal growth factor receptor 2
*JGCT*: Juvenile granulosa cell tumour
*LHRH*: Luteinizing hormone-releasing hormone
*NGS*: Next-generation sequencing
*OSCCHT*: Ovarian small cell carcinoma of hypercalcaemic type
*TGFβ*: Transforming growth factor β
*TSO500*: TruSight Oncology 500
